# Activation and modulation of the host response to DNA damage by an integrative and conjugative element

**DOI:** 10.1128/jb.00462-24

**Published:** 2025-01-23

**Authors:** Saria McKeithen-Mead, Mary E. Anderson, Alam García-Heredia, Alan D. Grossman

**Affiliations:** 1 Department of Biology, Massachusetts Institute of Technology 311383 , Cambridge, Massachusetts, USA; Geisel School of Medicine at Dartmouth, Hanover, New Hampshire, USA

**Keywords:** horizontal gene transfer, mobile genetic elements, integrative and conjugative elements, conjugation, DNA damage, SOS response, RecA, *Bacillus subtilis*, ICE*Bs1*

## Abstract

Mobile genetic elements help drive horizontal gene transfer and bacterial evolution. Conjugative elements and temperate bacteriophages can be stably maintained in host cells. They can alter host physiology and regulatory responses and typically carry genes that are beneficial to their hosts. We found that ICE*Bs1*, an integrative and conjugative element (ICE) of *Bacillus subtilis*, inhibits the host response to DNA damage (the SOS response). Activation of ICE*Bs1* before DNA damage reduced host cell lysis that was caused by SOS-mediated activation of two resident prophages. Further, activation of ICE*Bs1* itself activated the SOS response in a subpopulation of cells, and this activation was attenuated by the functions of the ICE*Bs1* genes *ydcT* and *yddA* (now *ramT* and *ramA*; *ram* for RecA modulator). Double-mutant analyses indicated that RamA functions to inhibit and RamT functions to both inhibit and activate the SOS response. Both RamT and RamA caused a reduction in RecA filaments, one of the early steps in activation of the SOS response. We suspect that there are several different mechanisms by which mobile genetic elements that generate single-stranded DNA (ssDNA) during their life cycle inhibit the host SOS response and RecA function, as RamT and RamA differ from the known SOS inhibitors encoded by conjugative elements.

**IMPORTANCE:**

Bacterial genomes typically contain mobile genetic elements, including bacteriophages (viruses) and integrative and conjugative elements, that affect host physiology. ICEs can excise from the chromosome and undergo rolling-circle replication, producing ssDNA, a signal that indicates DNA damage and activates the host SOS response. We found that following excision and replication, ICE*Bs1* of *B. subtilis* stimulates the host SOS response and that ICE*Bs1* encodes two proteins that limit the extent of this response. These proteins also reduce the amount of cell killing caused by resident prophages following their activation by DNA damage. These proteins are different from those previously characterized that inhibit the host SOS response and represent a new way in which ICEs can affect their host cells.

## INTRODUCTION

Horizontal gene transfer contributes to microbial evolution by allowing bacteria to acquire new genes and phenotypes through the transfer of DNA from a donor organism to a recipient. Horizontal gene transfer is often mediated by mobile genetic elements (MGEs), including bacteriophages and conjugative elements, and many bacterial genomes contain multiple MGEs. Functional or defective temperate phages are usually (but not always) found integrated in the bacterial genome. Conjugative elements include plasmids (extrachromosomal) and integrative and conjugative elements (ICEs) that reside integrated in the host genome. Both conjugative plasmids and ICEs encode conjugation machinery, a type IV secretion system, that is capable of contact-dependent transfer of element (and sometimes other) DNA from donor to recipient cells to generate transconjugants. Bacteriophages and conjugative elements can co-evolve with a bacterial host.

**Fig 1:**
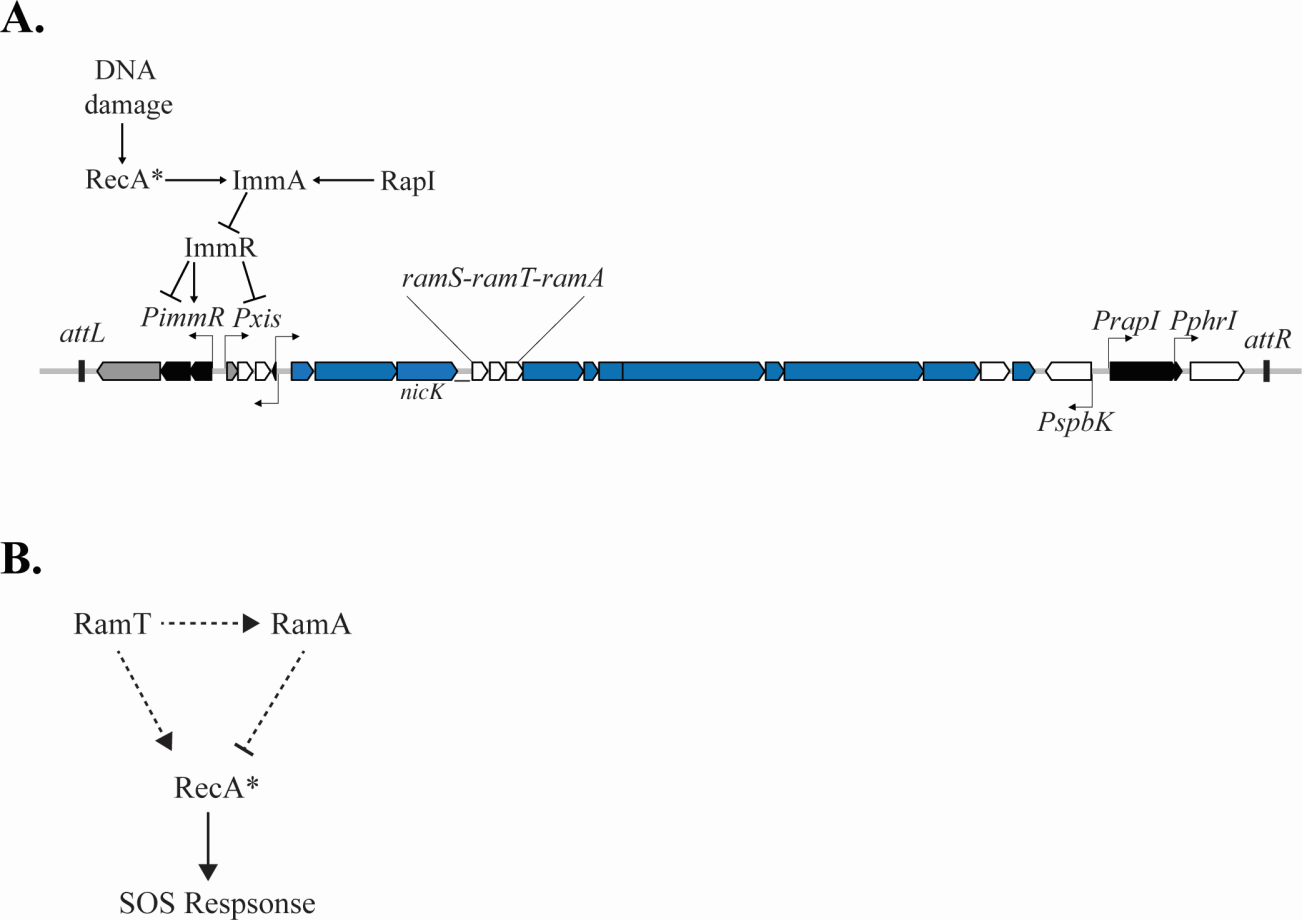
Gene map and regulation of ICE*Bs1* and model for the functions of RamT and RamA. (A) Map of ICE*Bs1*. The junctions between the ICE (~21 kb) and the chromosome are indicated as small rectangles at the left (*attL*) and right (*attR*) ends of the element. Genes are represented as rectangles with arrows at the end indicating the direction of transcription. Blue: genes that are required for conjugation and encode the type IV secretion system and the relaxasome; gray: genes needed for integration (*int* at the left end) and excision (*xis*); black: genes involved in regulation, including *immA* (protease), *immR* (repressor), and *rapI-phrI* (quorum-sensing regulation); white: genes not required for conjugation, including cargo genes, genes of unknown function, and *ramS*, *ramT*, and *ramA* (*ram* for RecA modulator) (this work; previously *ydcS*, *ydcT*, and *yddA*, respectively). Lines with arrows above and below the gene map indicate promoters and the direction of transcription. The major promoter that drives transcription of the conjugation genes is P*xis* (2, 3). A simplified cartoon of regulation is depicted above. ImmR represses transcription from P*xis*, and both activates and represses transcription from P*immR* (3). When integrated in the chromosome, P*xis* is repressed by ImmR. P*xis* expression is de-repressed when ImmR is cleaved by the anti-repressor and protease ImmA (9), which is activated by the RecA-dependent DNA damage response or RapI. RapI is inhibited by the ICE*Bs1*-encoded peptide PhrI (not depicted) (2). (**B**) Model for the functions of RamT and RamA. RamA functions to inhibit, and RamT functions to both inhibit and activate the host SOS response through RecA, either directly or indirectly. RamA acts as an inhibitor of RecA. RamT both activates and inhibits RecA, and inhibition is through stimulating or enabling RamA repression. Dotted lines indicate that these interactions could be direct or more likely indirect.

ICE*Bs1* is an integrative and conjugative element of *Bacillus subtilis* (1, 2). It is regulated by cell-cell signaling and activated by the host SOS response to DNA damage (Fig. 1A) (2). As with other ICEs, ICE*Bs1* excises from the chromosome, and the circular ICE DNA is nicked, unwound, and undergoes rolling-circle replication (3–7). Replication is important in the original host (donor) for maintenance of the element during cell growth (6) and in transconjugants to allow for re-repression and integration into the genome (5, 8). DNA unwinding and rolling-circle replication generate the linear single-stranded DNA (ssDNA) that is transferred from donor to recipient during conjugation (4, 6).

In many bacterial species, including *B. subtilis*, the presence of ssDNA is typically a signal of DNA damage and can initiate the host SOS response (10, 11). Filaments of RecA form on the ssDNA, activating RecA for its roles in gene regulation and homologous recombination (12–16). Because rolling-circle replication of ICE*Bs1* generates ssDNA that could trigger the SOS response, we postulated that the element might have genes that reduce this potential response, perhaps analogous to *psiB* of the family of F plasmids of *Escherichia coli* (17–19). The *psiB* gene product reduces the SOS response in transconjugants by binding to RecA and inhibiting formation of RecA filaments on the ssDNA (19–22).

We found that ICE*Bs1* inhibits the host SOS response to DNA damage in host (donor) cells. When activated, ICE*Bs1* inhibited DNA damage-induced host killing by the temperate phage SPβ and the defective temperate phage PBSX. Two genes in ICE*Bs1*, *ydcT* and *yddA* (now called *ramT* and *ramA*; *ram* for RecA modulator), were involved: both RamA and RamT functioned to inhibit, and RamT also functioned to activate the host SOS response (Fig. 1B). These proteins affected the accumulation of RecA filaments, an essential process in the normal cellular response to DNA damage. Additionally, activation of ICE*Bs1* itself stimulated the SOS response in a subpopulation of cells, and *ramT* and *ramA* functioned to limit this activation. *ramT* and *ramA* are conserved in some ICEs and do not appear to be related to genes on other conjugative elements that are known to inhibit the SOS response. We suspect that many ICEs have genes that modulate host responses to DNA damage, perhaps in both donor and recipient cells, and that there are likely a variety of different mechanisms used.

## RESULTS

### ICE*Bs1* reduces cell lysis caused by ultraviolet irradiation

In many species of bacteria, irradiation with ultraviolet (UV) light causes DNA damage and a global regulatory response, the SOS response (23). This response can result in cell lysis, mediated, at least in part, by de-repression of resident prophage. We monitored effects of ICE*Bs1* in *B. subtilis* on the DNA damage response by measuring cell growth and phage-mediated lysis following UV irradiation. Briefly, cells were grown in defined minimal medium to mid-exponential phase and exposed to UV light, and cell density (OD_600_) was monitored for several hours (Materials and Methods). Where indicated, ICE*Bs1* was activated by adding xylose to cause expression of P*xyl*-*rapI*. RapI activates ICE*Bs1* (Fig. 1A) by causing cleavage of the element-encoded repressor ImmR by the element-encoded protease ImmA (3, 9, 24).

**Fig 2:**
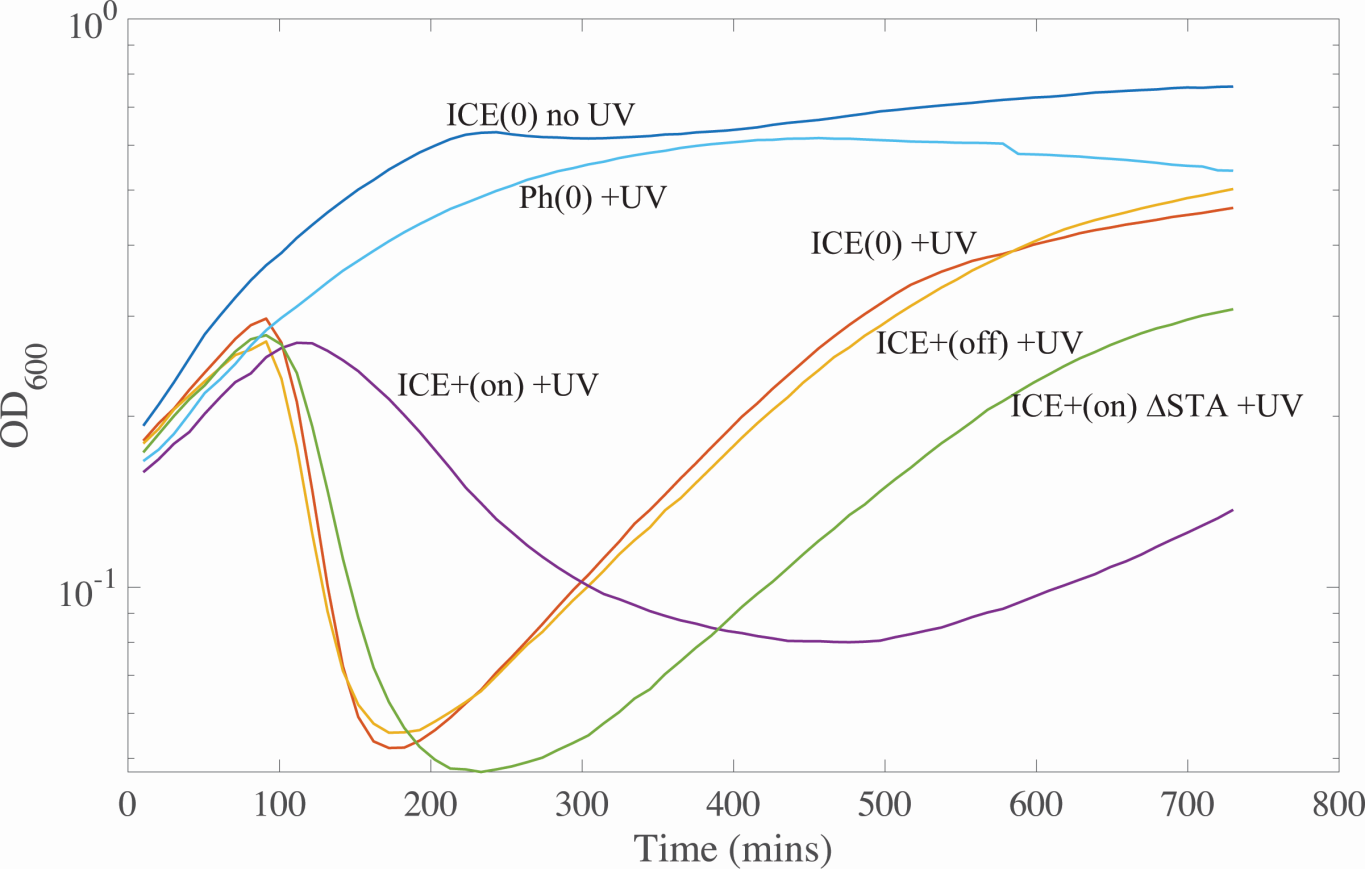
Expression of ICE*Bs1* prior to UV damage protects *B. subtilis* from phage-mediated lysis. Strains were grown at 37°C in minimal medium with 1% L-arabinose and grown to mid-exponential phase (OD_600_ ~0.2). Where indicated (ICE [on]), ICE*Bs1* was activated by inducing expression of *rapI* (from P*xyl*-*rapI*) with addition of 1% D-xylose 15 min prior to UV irradiation. Where indicated (+UV), cells were irradiated with UV light (Materials and Methods) and then grown by shaking in a 96-well plate. Growth was followed using a plate reader. OD_600_ is plotted vs time after irradiation. Strains containing ICE*Bs1* [ICE(+), SAM248] or cured of ICE*Bs1* [ICE(0), SAM591] were irradiated (+UV) or not (no UV). Ph(0) indicates a strain that is missing the two temperate phages, SPβ and PBSX, but contains ICE*Bs1* (SAM614), activated for this experiment. ICE ΔSTA indicates cells that contain the ICE*Bs1* mutant that is missing *ramS*, *ramT*, and *ramA* (SAM601). Data from a representative experiment are shown. Similar results were obtained from at least three independent experiments.

As expected, treatment with UV light caused cell lysis. Cultures of cells containing an integrated, repressed ICE*Bs1* (ICE+ [off], SAM248) or cells without ICE*Bs1* [ICE(0), SAM591] grew normally without UV irradiation but had a precipitous drop in optical density (OD) between 100 and 200 min after UV irradiation, indicating cell lysis (Fig. 2). Following the drop in culture OD, there was a resumption of cell growth indicated by the increase in OD. Cells that were missing both SPβ and PBSX [Ph(0), SAM614] had no detectable drop in OD following UV irradiation (Fig. 2), indicating that the temperate phages SPβ and PBSX were responsible for cell lysis following UV irradiation.

Activation of ICE*Bs1* 15 min prior to UV irradiation (ICE+ [on], SAM248) caused a delay in cell lysis, a smaller drop in the OD of the culture, and a delay in the resumption of exponential growth (Fig. 2). Based on these results, we conclude that activation of ICE*Bs1* conferred partial protection against phage-mediated lysis caused by the SOS response. As shown below, this is due to genes in ICE*Bs1* that modulate the host SOS response to DNA damage.

### A region of ICE*Bs1* that is necessary for ICE*Bs1-*mediated inhibition of DNA damage-induced cell lysis

Activation of ICE*Bs1* leads to robust expression of the polycistronic message produced from the promoter P*xis* (2, 3), including expression of genes involved in excision, replication, and conjugation (Fig. 1A). Because the observed inhibition of cell lysis required activation of ICE*Bs1*, the genes involved were likely expressed from P*xis* and not expressed when the element is integrated in the host chromosome and most element genes are repressed. We decided to initially focus on three genes with unknown function: *ramS*, *ramT*, and *ramA*, located downstream of *nicK* (encoding the element relaxase) (Fig. 1A).

We deleted the *ramS*, *ramT*, and *ramA* gene cluster (∆STA) in ICE*Bs1* and measured the effects of this mutant element on cell growth following UV irradiation, essentially as described above. In contrast to ICE*Bs1*+, we found that ICE*Bs1* ΔSTA (ICE+ ∆STA, SAM601) did not confer protection against cell lysis and following UV irradiation (Fig. 2). There was a precipitous drop in culture density, similar to or perhaps a bit more severe than that observed for cells without activation of ICE*Bs1* (Fig. 2). The apparent increase in sensitivity to UV irradiation compared to cells without ICE*Bs1* or with an uninduced ICE*Bs1* might be due to production of ICE*Bs1* ssDNA during rolling-circle replication following induction of the element (25–27).

### Activation of ICE*Bs1* stimulates the host SOS response

**Fig 3:**
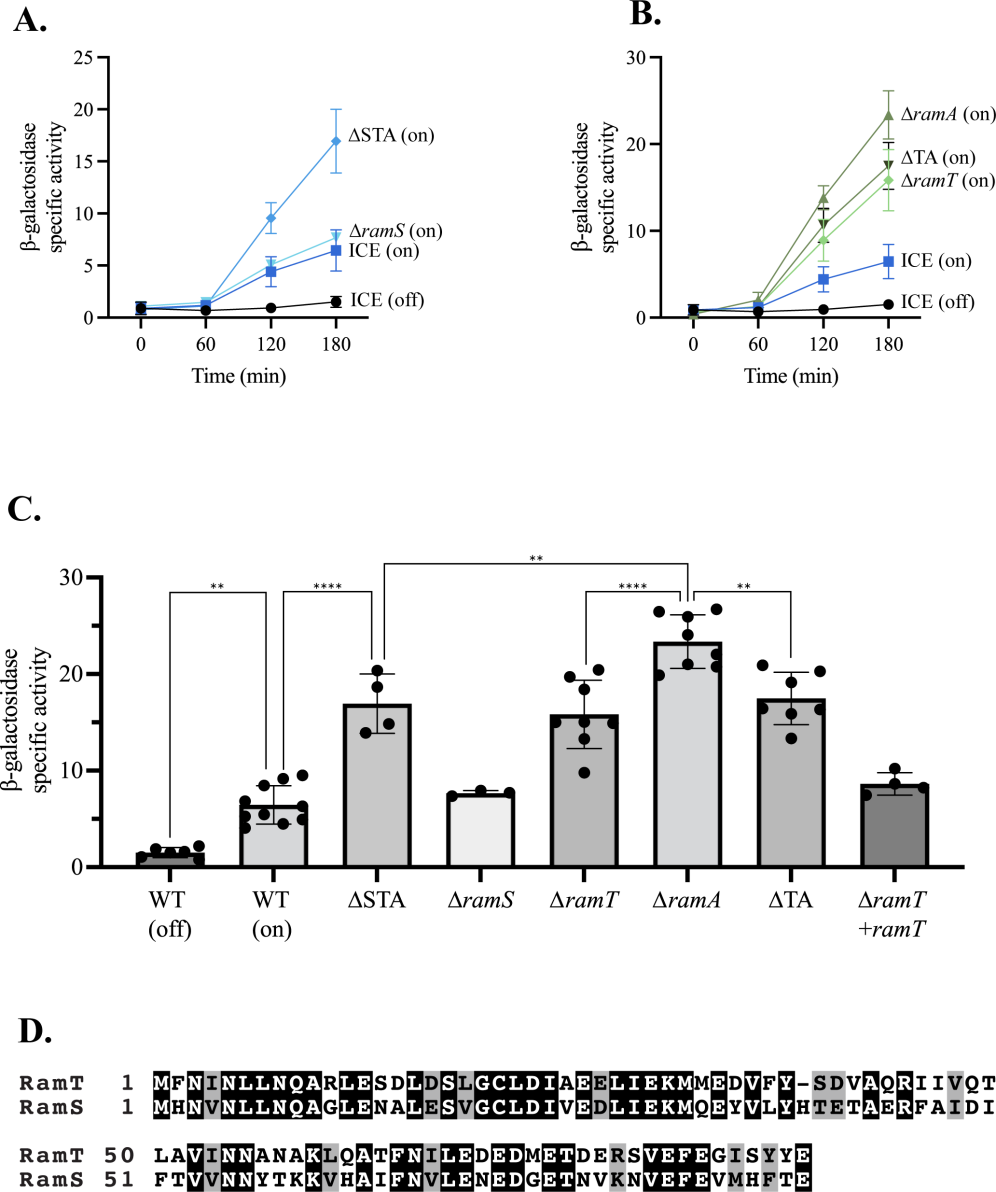
Effects of ICE*Bs1* and *ramSTA* on the host SOS response. Strains were grown at 37°C in minimal medium with 1% L-arabinose. ICE*Bs1* was activated where indicated by inducing *rapI* (P*xyl*-*rapI*) with the addition of 1% D-xylose when cells reached an OD of ~0.2. All strains have activated ICE*Bs1* except for ICE (off) samples. Samples were taken at indicated times, and β-galactosidase-specific activity was measured. All strains contain either wild-type ICE*Bs1* (ICE) or ICE*Bs1* with either single-gene deletions (as indicated) or multiple-gene deletions, as indicated: ∆TA (∆*ramT-A*) and ∆STA (∆*ramS-T-A*). Data represent the mean of at least three independent biological replicates, and error bars depict standard deviation. (**A and B**) β-Galactosidase-specific activity vs time after activation of ICE*Bs1*: ICE (off) = AGH270 (black circles), ICE (on) = AGH270 (squares). (A) ∆*ramS* = AGH241 (inverted triangles), ∆STA = AGH239 (diamonds). (B) ∆*ramT* = AGH207 and AGH353 (diamonds), ∆TA = AGH209 and AGH395 (inverted triangles), ∆*ramA* = AGH296 (triangles). (**C**) β-Galactosidase-specific activity 3 h after activation of ICE*Bs1*. Data are the same as presented in panels A and B. ∆*ramT + ramT* = AGH615. One-way analysis of variance: **P < 0.01, ****P < 0.001. Data that are not statistically different include ICE (on) vs ∆*ramS* and ∆STA vs ∆*ramT* vs ∆TA. (**D**) Protein sequence alignment of RamT and RamS. RamT and RamS protein sequences were aligned using National Center for Biotechnology Information Protein Basic Local Alignment Search Tool, and the graphic was generated using Boxshade. Identical residues are highlighted in black, and similar residues are highlighted in gray.

We found that activation of ICE*Bs1* stimulated the host SOS response (Fig. 3). We monitored the SOS response by measuring expression from the promoter of the SOS-inducible gene *yneA* fused to *lacZ* (P*yneA-lacZ*). *yneA* is repressed by LexA and de-repressed during the SOS response (16, 28). We found that expression of P*yneA-lacZ* increased 120 min following activation of ICE*Bs1* (Fig. 3A through C; ICE [on], AGH270). In contrast, expression was low in cells in which ICE*Bs1* had not been activated (Fig. 3A through C; ICE [off], AGH270). These results indicate that activation of ICE*Bs1* caused activation of the host SOS response. This is likely due to production of ICE*Bs1* ssDNA during rolling-circle replication following induction of the element. Activation of ICE*Bs1* that can excise from the chromosome does not normally cause cell death (29, 30), indicating that stimulation of the SOS response by ICE*Bs1* is not sufficient to activate the prophages SPβ and PBSX.

### *ramT* and *ramA* function to inhibit the host SOS response

We found that deletion of *ramS*, *ramT*, and *ramA* (∆STA) from ICE*Bs1* caused an increase in the SOS response following activation of the element (Fig. 3A and C; ∆STA; AGH239). By 3 h after activation, there was approximately twice as much β-galactosidase activity from P*yneA-lacZ* in cells with the ∆STA mutation compared to that from the wild type (Fig. 3A and C). These results indicate that the host SOS response was somehow reduced by one or more of the genes *ramS*, *ramT*, and *ramA*. Although RamS and RamT are 52.27% identical and 72.73% similar in a pairwise sequence alignment (Fig. 3D), we found that their functions are not redundant (see below).

#### ramS

Loss of *ramS* had little to no effect on activation of the SOS response (Fig. 3A and C); that is, expression of P*yneA-lacZ* was virtually indistinguishable in strains with ICE*Bs1* wild type (AGH270) or ICE*Bs1* ∆*ramS* (AGH241) following activation of the element. Based on these results, we focused on *ramT* and *ramA*.

#### *ramT* and *ramA*

We found that deletion of either *ramT* (AGH207/AGH353) or *ramA* (AGH296) caused an increase in expression of P*yneA-lacZ* relative to that caused by activation of wild-type ICE*Bs1* (Fig. 3B and C). Expression in the *ramA* mutant was consistently greater than that in the *ramT* mutant (Fig. 3B and C). Because the phenotype of each mutant was similar, and loss of *ramA* had a larger effect than loss of *ramT*, it was possible that the mutation in *ramT* was polar on *ramA*. This was not the case. We used a complementation test to determine polarity. We cloned ICE*Bs1* genes from *immA* (second gene from the left in Fig. 1A) through *ramT*, including the main promoter P*xis* (Fig. 1) and integrated these into an ectopic site (*cgeD*) in the chromosome. The inclusion of these genes and regulatory elements of ICE*Bs1* should ensure expression of *ramT* that is similar to that in the native element. As expected, the phenotype of cells containing ICE*Bs1* ∆*ramT* and the ectopic copy of *ramT*+ was similar to that caused by wild-type ICE*Bs1* (Fig. 3C); that is, expression of P*yneA-lacZ* was reduced, indicating that the effects of ∆*ramT* were due to loss of *ramT* and not polar effects on *ramA*. Together, these results establish the genetic formalism that both RamA and RamT act as inhibitors of the host SOS response.

Expression of P*yneA-lacZ* in a *ramT ramA* (∆TA, AGH209/AGH395) double mutant was less than that in the *ramA* single mutant (Fig. 3B and C). This reduced expression caused by loss of *ramT* indicates that RamT is an activator (in a formal sense, irrespective of mechanism) of the host SOS response. In other words, the elevated SOS response in the *ramA* single mutant relative to the double mutant was due to the activating effect of *ramT*. Furthermore, the phenotype of the *ramT ramA* double mutant was similar to that of the *ramT* single mutant (Fig. 3B and C); that is, there was no discernable effect of *ramA* on the SOS response in the absence of *ramT*. This indicates that RamT likely functions in some way to stimulate or enable RamA to inhibit the SOS response and that RamA has no effect on the SOS response without RamT. This could be by activating or working together with RamA or by affecting a cellular process that enables RamA to inhibit the SOS response.

To summarize the genetic interactions, the phenotypes of the *ramT* and *ramA* single and double mutants indicate that RamA functions to inhibit and RamT functions to both inhibit and activate the host SOS response, and that inhibition mediated by RamT is most likely through stimulating or enabling the repressive effects of RamA (Fig. 1B).

### The SOS response is induced in a subpopulation of cells with activated ICE*Bs1*

The P*yneA-lacZ* reporter indicates gene expression on a population level and does not distinguish whether some or all of the cells were undergoing an SOS response. To determine the fraction of cells in a population that were undergoing SOS, we used a P*yneA-mNeongreen* reporter and fluorescence microscopy to monitor the SOS response in single cells. We determined background fluorescence and used this to set a threshold for identifying cells that were expressing P*yneA-mNeongreen* (Materials and Methods).

**Fig 4:**
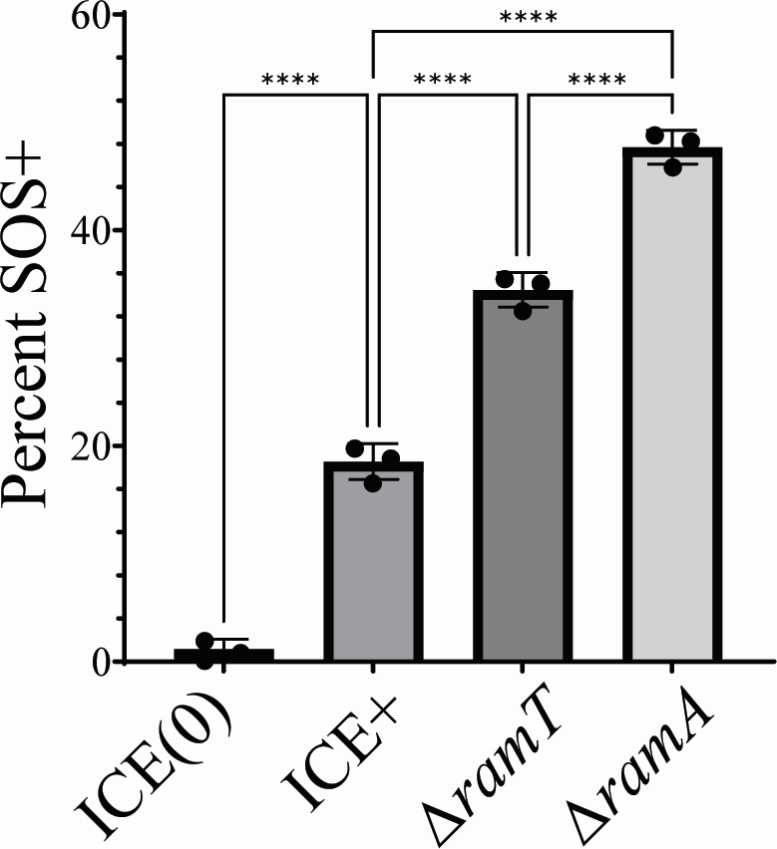
Activation of ICE*Bs1* induces the SOS response in a subpopulation of cells. Cells were grown at 37°C in minimal medium with 1% L-arabinose. ICE*Bs1* was activated by inducing *rapI* (P*xyl-rapI*) with the addition of 1% D-xylose when cells reached an OD_600_ of ~0.2. All strains contained P*yneA-mNeongreen* as an indicator of the SOS response. The fraction of the population expressing P*yneA*-*mNeongreen* 2 h after activation of ICE*Bs1* is shown. Data represent the mean of three independent biological replicates, and error bars depict standard deviation. ICE(0) = SAM362 (908 cells analyzed); ICE+ = SAM352 (1,120 cells analyzed); Δ*ramT* = SAM955 (1,146 cells analyzed); Δ*ramA* = SAM957 (1,179 cells analyzed). Statistical significance: one-way analysis of variance: ****P < 0.001.

In cells without ICE*Bs1*, <2% of cells were expressing P*yneA-mNeongreen*, indicative of the basal activation of SOS [Fig. 4; ICE(0), SAM362]. We found that 2 h after activation of ICE*Bs1*, approximately 18% of cells were fluorescent (Fig. 4; ICE+, SAM352), indicating that these cells had activated the SOS response. We cannot distinguish ICE-independent from ICE-dependent activation of SOS in the cells with ICE*Bs1* induced. However, approximately 10-fold more cells are activated for SOS following activation of ICEBs1 than in uninduced cells. These results indicate that activation of ICE*Bs1* stimulated the SOS response in a subpopulation of cells.

We found that *ramT* and *ramA* were involved in limiting the fraction of cells undergoing an SOS response following activation of ICE*Bs1*. Two hours after activation of ICE*Bs1* ∆*ramT*, approximately 35% of cells were expressing P*yneA-mNeongreen* (Fig. 4; ∆*ramT*, SAM955)*,* about twofold greater than that for wild-type ICE*Bs1*. Loss of *ramA* had a somewhat larger effect. Approximately 45%–50% of cells were expressing P*yneA-mNeongreen* 2 h after activation of ICE*Bs1* ∆*ramA* (Fig. 4; ∆*ramA*, SAM957) about 2.6-fold greater than that for wild-type ICE*Bs1* (Fig. 4; ICE+, SAM352), similar to the effects measured in the bulk population (Fig. 3B and C). Together, our results indicate that activation of ICE*Bs1* stimulates the SOS response in a subpopulation of cells and that *ramT* and *ramA* normally function to limit this activation.

### Formation of RecA filaments is inhibited by *ramT* and *ramA* during the SOS response

**Fig 5:**
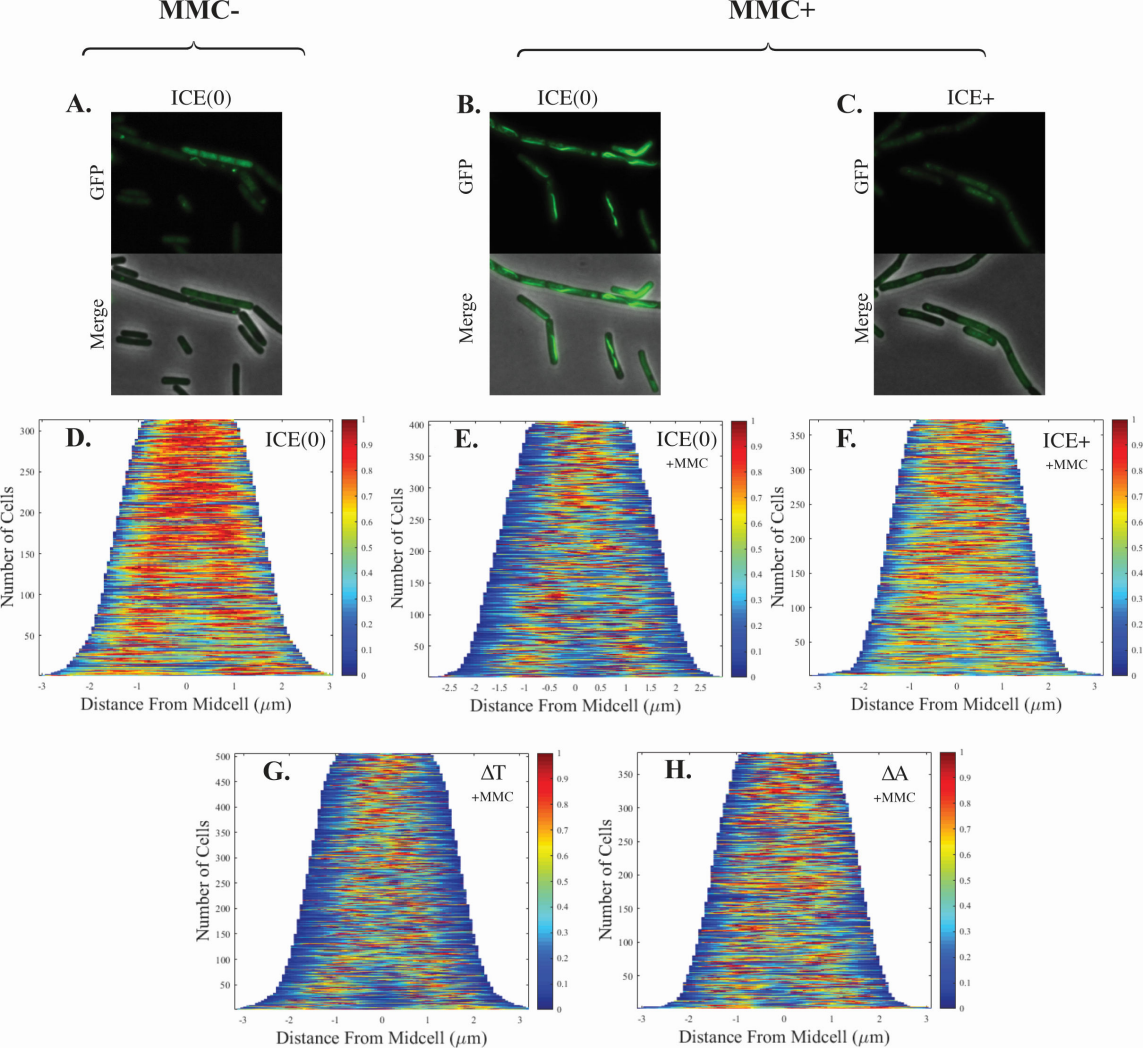
ICE*Bs1*-mediated inhibition of formation of RecA-GFP filaments. Strains were grown at 37°C in minimal medium with 1% L-arabinose to an OD_600_ of ~0.2, treated as indicated below, and samples were taken for microscopy to visualize RecA-GFP. Exponentially growing cells without MMC (**A and D**) and 15 min after treatment with MMC (+MMC; **B, C, and E–H**). ICE*Bs1* was activated (**C and F–H**) by inducing *rapI* (P*xyl*-*rapI*) with the addition of 1% D-xylose for 15 min before the addition of MMC. ICE(0) indicates strains that were cured of ICE*Bs1*. ICE+, ∆T, and ∆A indicate strains containing mini-ICE elements (deleted for all the genes downstream of *ramA*), which contain are otherwise either wild type, ∆*ramT*, or ∆*ramA*, respectively. At least three biological replicates with six frames for each condition were analyzed. Demographs are from 30 min after the addition of MMC. (**A–C**) Representative micrographs of RecA-GFP filament formation under different conditions. (**D–H**) Representative demographs of RecA-GFP filament formation. The mean fluorescence intensity of RecA-GFP is indicated by the color scale (right *y*-axis), and the localization within the cell (determined as distance from mid-cell) is represented on the *x-*axis. The cumulative number of cells analyzed is indicated on left *y*-axis. (**A, B, and D, E**) ICE*Bs1*(0) = SAM591, (**C and F**) WT ICE*Bs1* = SAM734, (**G**) ∆*ramT* = SAM1003, and (**H**) ∆*ramA* = SAM1004.

Binding of RecA to ssDNA following DNA damage leads to formation of RecA filaments along the ssDNA. We measured the levels and distribution of RecA-GFP in individual cells following treatment with mitomycin-C to induce the SOS response (Fig. 5A through C) and summarized these data in demographs (Fig. 5D through H). We used strains that were either missing ICE*Bs1* [ICE(0)] or cells that had a mini-ICE containing all of the genes from *attL-ramA* (Fig. 1A) but missing the genes downstream of *ramA*, including those encoding the mating machinery. This eliminated the ability of the element to transfer and allowed us to focus on our genes of interest (*ramS-A*) and those involved in element replication, which generates ssDNA. Cells were sorted by length, with small cells at the top of the demographs and longer cells that are nearing or undergoing cell division at the bottom. The colors indicate the mean fluorescence intensity of RecA-GFP (red for higher intensity, blue for lower intensity) at the corresponding distance from the mid-cell (*x*-axis). The cumulative number of cells analyzed for each condition is indicated on the *y*-axis, and the distance from the mid-cell is indicated on the *x*-axis.

In cells growing exponentially without ICE*Bs1* (and no mitomycin-C), RecA-GFP was distributed throughout the cell and did not form filaments or foci [Fig. 5A; ICE(0), no MMC, SAM591] similar to what has been described previously (31). In the demograph, this is represented by a general red signal across much of the length of the cell, typically centered around mid-cell in smaller cells and the cell quarters (which will become mid-cell following division) in larger cells [Fig. 5D; ICE(0), no MMC, SAM591].

Treatment of cultures with mitomycin C for 30 min resulted in the formation of RecA-GFP filaments in strains without ICE*Bs1* [Fig. 5B; ICE(0), +MMC, SAM591]. This is seen as the loss of much of the red throughout the cells and a more focused signal near the mid-cell (or the cell quarters), and a concomitant increase in blue in the demographs [Fig. 5E; ICE(0), +MMC, SAM591]. The mid-cell position is likely indicative of the location of the replisome and consistent with previous findings (31).

Strikingly, formation of RecA-GFP filaments was quite different in strains in which ICE*Bs1* had been activated for 15 min before treatment with mitomycin C (Fig. 5C and F; ICE+, +MMC, SAM734). The distribution of RecA-GFP was between that seen in ICE-cured cells without (Fig. 5D) and with mitomycin C (Fig. 5E). These results indicate that expression of ICE*Bs1* was inhibiting the formation of RecA-GFP filaments.

We found that both *ramT* and *ramA* were required for the ICE*Bs1*-mediated inhibition of RecA-GFP filament formation. The pattern of RecA-GFP filaments in a population of cells in which ICE*Bs1* ∆*ramT* (Fig. 5G; ∆T, +MMC, SAM1003) or ICE*Bs1* ∆*ramA* (Fig. 5H; ∆A, +MMC, SAM1004) had been activated for 15 min prior to MMC treatment was largely similar to that in cells without activation of ICE*Bs1* (Fig. 5E).

Together, our results indicate that *ramT* and *ramA* inhibit the cellular SOS response and that this inhibition is mediated, at least in part, by either directly or indirectly affecting formation of RecA filaments.

## DISCUSSION

Our work demonstrates that ICE*Bs1* modulates the host DNA damage (SOS) response by inhibiting accumulation of RecA filaments, either directly or indirectly. We found that activation of ICE*Bs1*, without external damaging agents, was sufficient to activate the SOS response in a subpopulation of cells and that the ICE*Bs1* products RamT and RamA attenuate this response by reducing the presence of RecA filaments. Further, activation of ICE*Bs1* prior to treatment with a DNA damaging agent also reduced the SOS response, reducing activation of resident temperate phages, thereby limiting phage-mediated cell death. This effect was also mediated by *ramT* and *ramA*. Our results show that RamA inhibits and RamT both inhibits and activates the host SOS response. Moreover, the inhibition mediated by RamT is likely achieved by stimulating inhibition by RamA (Fig. 1B), and RamA does not appear to be functional in the absence of RamT.

### RamT and RamA functions

We do not know the mechanism(s) by which RamT and RamA affect RecA filaments. The accumulation of RecA filaments is complex and involves several host proteins (reviewed in references [32, 33]). For example, ssDNA is bound by the host single-strand binding protein Ssb. Ssb inhibits RecA binding, apparently by competing for ssDNA (34–36). Ssb is also needed for recruitment of RecO in order to load RecA onto ssDNA (37–39). In these ways, Ssb has both positive and negative effects on assembly of RecA filaments. Additionally, RecA filaments are removed from DNA by the DNA translocase PcrA (40–43). It is possible that RamT and RamA bind directly to RecA, analogous to PsiB binding to RecA (19). However, we think this is unlikely, given the complicated phenotypes of the *ramT* and *ramA* mutants. Rather, we postulate that RamT and RamA modulate one of the several other proteins known to be involved in the accumulation of RecA filaments.

Our experiments were done with a population of “donor” cells, all of which contained the element. We suspect that the inhibition of SOS also occurs in transconjugants shortly after transfer of ICE*Bs1* to a new host. The DNA that is transferred by type IV secretion systems is linear ssDNA, a substrate for Ssb and indicator of DNA damage. After entry into a new host and the conversion of the linear ssDNA to circular dsDNA, *ramT* and *ramA* will be expressed from the strong element promoter P*xis*. We postulate that this expression will help limit the SOS response in transconjugants, before P*xis* is repressed and the element integrates into the chromosome.

We hypothesize that the ability of ICE*Bs1* to inhibit activation of the SOS response allows the element to remain activated with the potential to transfer to other cells for extended periods of time while reducing fitness costs to its host. Active rolling-circle replication produces a considerable amount of ssDNA bound by Ssb (25–27). This is typically a signal of DNA damage and causes activation of the SOS response and subsequent inhibition of cell division. Limiting the population of cells undergoing the SOS response likely allows many cells to continue to divide while the element is extrachromosomal and in multicopy. We suspect that inhibition of the SOS response is a conserved property of other ICEs that undergo robust rolling-circle replication.

Many of the *Bacillus* strains that contain ICE*Bs1* or ICE*Bs1*-like elements also harbor prophages (both functional and defective) that are activated by the SOS response, as is ICE*Bs1*. In our lab strains, the SOS response elicited by activation of ICE*Bs1* is not sufficient to activate the resident prophages SPβ and PBSX, even in the absence of *ramT* and *ramA*. However, activation of ICE*Bs1* prior to more robust DNA damage reduces activation of the resident prophages. If an element is activated at a lower threshold of DNA damage than the resident prophages, then this could help protect cells from phage-mediated killing. Further, there are likely other temperate phages that are more sensitive to DNA damage signals than SPβ or PBSX. We suspect that ICE-mediated inhibition of SOS likely protects cells from activation of those phages.

### Multiple ways ICE*Bs1* affects the physiology of host cells

Inhibition of RecA filaments and the SOS response is one of at least four ways in which ICE*Bs1* affects host cell physiology and/or fitness. The three other known ways include the following: (i) the ICE*Bs1* gene *devI* causes a delay in sporulation and biofilm formation when the element is active (excised from the chromosome with its genes expressed) and allows extra cell divisions to ICE*Bs1*-containing cells; this delay in development enables those cells to out-compete neighboring cells without the element (44); (ii) ICE*Bs1* has an abortive infection mechanism mediated by *spbK* that protects ICE*Bs1* host populations from predation by the phage SPβ (45); *spbK* is expressed independently of other ICE*Bs1* genes, even when the element is integrated in the host chromosome; (iii) the product of the ICE*Bs1* gene *yddJ* inhibits the activity of the cognate conjugation machinery in other cells; this “exclusion” mechanism limits excessive conjugative transfer and its potentially lethal effects on the cell envelope (46). Together these strategies play an important role in the transfer and maintenance of ICE*Bs1* and increase the competitive advantage of both the element and its host.

### Other modulators of the SOS response encoded by mobile genetic elements

ICE*Bs1* is not the only mobile genetic element that has genes for inhibiting the host SOS response. One of the best understood element-encoded inhibitors of the host SOS response is PsiB, encoded by the F plasmid of *E. coli* and other related conjugative plasmids. *psiB* is expressed in transconjugants, and its product binds to and inhibits RecA, thereby limiting the SOS response and activation of resident prophages (19, 21, 22, 47, 48).

Although RamT and RamA inhibit RecA filaments, they are not similar in sequence to PsiB or any other characterized protein. Further, PsiB appears to be expressed only in transconjugants (20, 22), whereas RamT and RamA are produced and act in donors, and we suspect also act in transconjugants. Conjugative plasmids do not undergo extensive rolling-circle replication in donors and hence produce a limited amount of ssDNA, likely obviating the need for expression of an anti-SOS function in donors. In contrasts, ICE*Bs1* undergoes robust rolling-circle replication and produces enough ssDNA to induce the SOS response. Further, RamT acts in both a positive and negative way on RecA filaments and is required for RamA to act. In these ways, the mechanisms by which RamT and RamA function are likely to be different from that of PsiB.

Some bacteriophages also have mechanisms to modulate the host SOS response and recombination systems. For example, bacteriophage lambda encodes the Redβ recombination system (*exo*, exonuclease; *bet*, strand-annealing protein; *gam*, RecBCD nuclease inhibitor), along with *orf* (recombination mediator) and *rap* (Holliday junction endonuclease). Together these proteins are sufficient to perform many of the host functions of DNA damage repair, including homologous recombination, recognition of SSB-bound ssDNA, and Holliday junction resolution (49–51). Though these genes, in large part, are there to complete a specific step in the lambda phage life cycle, some inhibit activation of the SOS response. Gam inhibits the activity of the exonuclease RecBCD (essential for double-strand break repair) when lambda switches from theta to rolling-circle replication (52, 53), a crucial point when lambda is most likely to activate the SOS response.

Some phages act directly on LexA to inhibit the host SOS response. For example, the tectiviral temperate phage GIL01 of *Bacillus thuringiensis* encodes a small protein (gp7) that forms a stable complex with LexA, enhancing binding of LexA to SOS boxes and inhibiting RecA-mediated autocleavage required to activate the SOS response (54). GIL01 dampens the host SOS response and activation of other co-resident prophages to ensure that it is able to become active and replicate before the host activates the SOS response and de-represses other phages (55–57).

Determining how ICEs modulate the physiology of their hosts is critical for understanding the complex relationships between different mobile elements within a given host. These interactions are continually evolving, and conjugative elements have multiple ways to help protect their bacterial hosts from predation by phages, including modulating the SOS response.

## MATERIALS AND METHODS

### Media and growth conditions

Cells were grown in lysogeny broth (LB) medium (usually for strain construction) or S750 defined minimal medium (58). For experiments, all strains were colony purified from frozen (−80°C) glycerol stocks on LB agar plates with the appropriate antibiotics, and cells from a single colony were inoculated into S750 minimal medium with 1% L-arabinose (wt/vol) as the carbon source and required amino acids (40 µg/mL phenylalanine and 40 µg/mL tryptophan). Cells were grown to mid-exponential phase, then diluted to an OD_600_ of 0.025 and grown at 37°C with shaking until an OD_600_ of ~0.2 was obtained. ICE*Bs1* was activated by adding 1% D-xylose to induce expression of Pxyl-*rapI*. RapI causes the ICE*Bs1*-encoded protease ImmA to cleave the repressor ImmR, thereby de-repressing ICE*Bs1* gene expression (Fig. 1A) (3, 9, 24). Antibiotics were used at the following concentrations: 5 µg/mL kanamycin, 10 µg/mL tetracycline, 100 µg/mL spectinomycin, 5 µg/mL chloramphenicol, and a combination of erythromycin at 0.5 µg/mL and lincomycin at 12.5 µg/mL to select for macrolide-lincosamide-streptogramin (*mls*) resistance.

### Bacterial strains and alleles

Strains were constructed by natural transformation. All strains (Table 1) are derivatives of JH642 (AG174 [59]) and contain *trpC* and *pheA* mutations (not indicated). Strains cured of ICE*Bs1* are indicated as ICE*Bs1*^0^. Strains containing either *amyE*::[(P*xyl*-r*apI*) *spc*] (60) or *lacA*::[(P*xyl-rapI*) *tet*] (6) were used to activate expression of ICE*Bs1* (following addition of xylose). Most ICE*Bs1* strains contained a kanamycin-resistance gene [Δ(*rapI-phrI*)*342*::*kan*] (2) or a derivative to allow selection for the element. The *recA*:{(*recA-gfp*) *spc*} (31), the Ph(0) ∆PBSX::*lox* ∆SPβ::*lox* (61), the P*yneA-mNeongreen* (8), and the P*yneA-lacZ* (62) (obtained from the Bacillus Genetic Stock Center) alleles have all previously been described.

**TABLE 1:** *B. subtilis* strains used^*a*^

Strain	Relevant genotype
AGH207	ICE*Bs1* [Δ*ramT*::*lox* Δ(*rapI-phrI*)*342*::*kan*] Δ*lacA*::{(P*xyl-rapI*) *spc*} ∆*amyE*::{(P*yneA-lacZ*) *cat*}^*b*^
AGH209	ICE*Bs1* [Δ*ramT-A*::*lox* Δ(*rapI-phrI*)*342*::*kan*] Δ*lacA*::{(P*xyl-rapI*) *spc*} ∆*amyE*::{(P*yneA-lacZ*) *cat*}
AGH239	ICE*Bs1* [Δ*ramS-A*::*lox* Δ(*rapI-phrI*)*342*::*kan*] Δ*lacA*::{(P*xyl-rapI*) *spc*} ∆*amyE*::{(P*yneA-lacZ*) *cat*}
AGH241	ICE*Bs1* [Δ*ramS*::*lox* Δ(*rapI-phrI*)*342*::*kan*] Δ*lacA*::{(P*xyl-rapI*) *spc*} ∆*amyE*::{(P*yneA-lacZ*) *cat*}
AGH270	ICE*Bs1* [Δ(*rapI-phrI*)*342*::*kan*] Δ*lacA*::{(P*xyl-rapI*) *tet*} ∆*amyE*::{(P*yneA-lacZ*) *cat*}
AGH296	ICE*Bs1* [Δ*ramA*::*mls* Δ(*rapI-phrI*)*342*::*kan*] Δ*lacA*::{(P*xyl-rapI*) *tet*} ∆*amyE*::{(P*yneA-lacZ*) *cat*}
AGH353	ICE*Bs1* [Δ*ramT*::*lox* Δ(*rapI-phrI*)*342*::*kan*] Δ*lacA*::{(P*xyl-rapI*) *tet*} ∆*amyE*::{(P*yneA-lacZ*) *cat*}
AGH395	ICE*Bs1* [Δ*ramT-A*::*lox* Δ(*rapI-phrI*)*342*::*kan*] Δ*lacA*::{(P*xyl-rapI*) *tet*} ∆*amyE*::{(P*yneA-lacZ*) *cat*}
AGH462	ICE*Bs1*^0^ ∆*cgeD*::[ICE*Bs1* ∆*int* ∆(*conQ-attR*)::*cat*]
AGH606	ICE*Bs1*^0^ Δ*cgeD*::[ICE*Bs1* Δ*int oriT*^-^ Δ(*yddA-attR*)::*spc*]^*c*^
AGH615	ICE*Bs1* [Δ*ramT*::*lox* Δ(*rapI-phrI*)*342*::*kan*] Δ*lacA*::{(P*xyl-rapI*) *tet*} ∆*amyE*::{(P*yneA-lacZ*) *cat*} ∆*cgeD*::[ICE*Bs1* ∆*int oriT*^-^ Δ(*yddA-attR*)::*spc*]^*c*^
SAM248	ICE*Bs1* [Δ(*rapI-phrI*)*342*::*kan*] Δ*lacA*::{(P*xyl-rapI*) *tet*} *recA*:{(*recA-gfp*) *spc*}^*d*^
SAM352	ICE*Bs1* [Δ(*rapI-phrI*)*342*::*kan*] Δ*amyE*::{(P*yneA-mNeongreen*) *spc*} Δ*lacA*::{(P*xyl*-*rapI*) *tet*}^*d*^
SAM362	ICE*Bs1*^0^ Δ*amyE*::{(P*yneA-mNeongreen*) *spc*} Δ*lacA*::{(P*xyl-rapI*) *tet*}^*e*^
SAM591	ICE*Bs1*^0^ Δ*lacA*::{(P*xyl-rapI*) *tet*} *recA*:{(*recA-gfp) spc*}
SAM601	ICE*Bs1* [Δ*ramS-ramA* Δ(*rapI-phrI*)*342*::*kan*] Δ*lacA*::{(P*xyl-rapI*) *tet*} *recA*:{(*recA-gfp*) *spc*}
SAM614	ICE*Bs1* [Δ(*rapI-phrI*)*342*::*kan*] Δ*lacA*::{(P*xyl-rapI*) *tet*} *recA*:{(*recA-gfp*) *spc*} ΔPBSX::*lox* ΔSPβ::*lox*^*f*^
SAM734	ICE*Bs1* [Δ(*yddB-yddM*)::*kan*] Δ*lacA*::{(P*xyl-rapI*) *tet*} *recA*:{(*recA-gfp*) *spc*}
SAM955	ICE*Bs1* [Δ*ramT*::*lox* Δ(*rapI-phrI*)*342*::*kan*] Δ*amyE*::{(P*yneA- mNeongreen*) *spc*} Δ*lacA*::{(P*xyl-rapI*) *tet*}
SAM957	ICE*Bs1* [Δ*ramA*::*lox* Δ(*rapI-phrI*)*342*::*kan*] Δ*amyE*::{(P*yneA- mNeongreen*) *spc*} Δ*lacA*::{(P*xyl-rapI*) *tet*}
SAM1003	ICE*Bs1* [Δ*ramT* Δ(*yddB-yddM*)::*kan*] Δ*lacA*::{(P*xyl-rapI*) *tet*} *recA*:{(*recA*-*gfp*) *spc*}
SAM1004	ICE*Bs1* [Δ(*yddA-yddM*)::*kan*] Δ*lacA*::{(P*xyl*-*rapI*) *tet*} *recA*:{(*recA*-*gfp*) *spc*}

^*a*^ All strains are derived from JH642 (59) and contain *trpC2* and *pheA1* alleles (not indicated in the relevant genotypes).

^*b*^ A strain with ∆*amyE*::{(P*yneA-lacZ*) *cat*} (62) was obtained from the Bacillus Genetic Stock Center.

^*c*^ Version of ICE*Bs1* integrated at *cgeD* used for complementation. DNA from ICE*Bs1* extends from *immA* through *ramT* and has mutations that inactivate *oriT*. In this way, expression of *ramT* is controlled as it would be in wild-type ICE*Bs1*, except that the element is unable to excise and to initiate rolling-circle replication from *oriT*. Strain AGH615 contains both the ∆*ramT::lox* allele and the complementation construct and was used in the complementation test.

^*d*^ *recA*:{(*recA-gfp*) *spc*} allele (31).

^*e*^ Δ*amyE*::{(P*yneA-mNeongreen*) *spc*} allele (8).

^*f*^ ∆PBSX::*lox* ΔSPβ::*lox* allele (61).

#### Δ(*ramS-ramA*)

The three genes *ramS*, *ramT*, and *ramA* were deleted in two steps, first by integrating a plasmid and then by screening for loss of the integrated plasmid and the presence of the deletion allele. A fragment from ~500 bp upstream of *ramS* to the first 12 bp of *ramS* and a second fragment from the last 42 bp of *ramA* (to maintain the start of *conB*, which overlaps the end of *ramA*) to ~500 bp downstream of *ramA* were amplified by PCR and assembled into pCAL1422 ([7], a plasmid that contains *E. coli lacZ*) by isothermal assembly (63). The resulting plasmid pELC1582 was integrated into the chromosome by single-crossover recombination. Transformants were screened for loss of *lacZ*, indicating loss of the integrated plasmid, and PCR was used to identify a clone containing the Δ(*ramS ramT ramA*) allele (indicated as ∆*ramS-ramA* or ∆STA).

#### Δ*ramS*::*lox*, Δ*ramT*::*lox*, Δ*ramA*::*lox*, and Δ*ramT-ramA*::*lox*

Deletions of individual genes and the *ramT-ramA* double deletion with *lox* insertions were made by introducing an antibiotic resistance gene (*cat*) flanked by *lox* sites into each gene followed by Cre-mediated recombination to remove the antibiotic resistance gene and leaving a *lox* insertion in place of the coding sequence. Regions from about ~1 kb upstream to ~3 bp in each gene and from the stop codon to ~1 kb downstream of each gene, except for *ramA*, were amplified by PCR. For *ramA*, 28 bp of the 3′-end of the gene was included to maintain the *conB* open reading frame. For the *ramT-ramA* double deletion, a region from ~1 kb upstream of *ramT* to 4 bp into *ramT* and a region from the last 40 bp at the 3′-end of *ramA* to ~1 kb downstream of *ramA* were used. The *cat* cassette from pGemcat (64) was amplified with primers that added *lox71* and *lox66* sites. For each gene, the PCR fragments from upstream and downstream sequences were assembled on either side of *cat* (with the *lox* sites) using linear isothermal assembly (63). The assembled fragment was integrated into the chromosome by double-crossover and selecting for resistance to chloramphenicol. *cat* was removed by Cre-mediated recombination between the *lox* sites following introducing (by transformation) of a temperature-sensitive plasmid expressing the Cre recombinase (pSAM097). Strains were cured of pSAM097 by shifting cells to a non-permissive temperature and allowing for growth without selection for the plasmid. Diagnostic PCR and sequencing were used to confirm each allele.

#### Δ*ramA*::*mls*

The *ramA* deletion-insertion replaced most of the open reading frame of *ramA*, leaving intact the first 10 bp and the last 40 bp of the open reading frame, with an antibiotic resistance cassette (*mls*) using linear isothermal assembly. Regions flanking ~1 kb upstream and downstream of these boundaries and the *mls* cassette from pDG795 (65) were amplified by PCR, fused with linear isothermal assembly (63) and used for transformation.

#### *ramT* complementation

We used a complementation test to determine if ∆*ramT*::*lox* was polar on *ramA*. To complement the *ramT* mutation, we introduced *ramT* at an ectopic site in the chromosome using a truncated version of ICE*Bs1* to maintain proper expression from the major ICE*Bs1* promoter, P*xis*. Construction of this truncated ICE*Bs1* (~6.6 kb) was done in two steps.

First, four DNA fragments were assembled using linear isothermal assembly (63). We amplified ~1 kb fragments of the 5′ and 3′ ends of *cgeD*, the chromosomal gene into which the complementation construct was integrated; a 3,250 bp fragment that included ICE*Bs1* genes between *int* and *conQ*, including only the first 164 bp of the *int* coding region and the first 540 bp of *conQ*, and *cat* (chloramphenicol resistance) from pGemcat (64). The assembled fragments were transformed into a wild-type strain lacking ICE*Bs1* [ICE*Bs1*(0)], generating the strain AGH462 [ICE*Bs1*(0) Δ*cgeD*::[ICE*Bs1* ∆*int* ∆(*conQ-attR*)::*cat*]].

In the second step, another four DNA fragments were amplified and assembled. We amplified the remaining 903 bp of *conQ* through the intergenic region between *ramT* and *ramA* (106 bp after *ramT* , leaving out *ramA*), using genomic DNA from JMJ646 (44). ICE*Bs1* in JMJ646 contains a mutant *oriT* (*oriT*^−^) that is non-functional but retains a functional *nicK* with synonymous codons overlapping *oriT* (44). The absence of a functional *oriT* prevents autonomous rolling-circle replication of the element, which would be lethal in cells with ICE*Bs1* unable to excise from the chromosome. The *spc* antibiotic resistance cassette from pUS19 (66) and ~1 kb of DNA 5′ (in ICE*Bs1*) and 3′ (*cgeD*) were also amplified. These fragments were combined using linear isothermal assembly (63) and introduced into ICE*Bs1* at *cgeD* in strain AGH462 to generate strain AGH606 [ICE*Bs1*(0) Δ*cgeD*::[ICE*Bs1* Δ*int oriT*^-^ Δ(*yddA-attR*)::*spc*]]. This allele was transformed into AGH395 (ICE*Bs1* [Δ(*ramT-A*)::*lox* Δ(*rapI-phrI*)*342*::*kan*] Δ*lacA*::{(P*xyl-rapI*) *tet*} ∆*amyE*::{(P*yneA-lacZ*) *cat*}) to generate strain AGH615 (ICE*Bs1* [Δ*ramT*::*lox* Δ(*rapI-phrI*)*342*::*kan*] Δ*lacA*::{(P*xyl-rapI*) *tet*} ∆*amyE*::{(P*yneA-lacZ*) *cat*} ∆*cgeD*::[ICE*Bs1* ∆*int oriT*^-^ Δ(*yddA-attR*)::*spc*]).

#### Mini-ICE strains for RecA-GFP localization

Mini-ICE elements were constructed by deleting *yddB-yddM*, either with or without *ramA* or *ramT*, and introducing a kanamycin resistance cassette. For the wild-type mini-ICE, regions from ~1 kb upstream of *yddB* to the stop codon of *ramA* and from the last 5 bp of *yddM* to ~1 kb downstream of *yddM* were used. For the ∆*ram*A mini-ICE, regions from ~1 kb upstream of *ramA* to 24 bp downstream of *ramT* and from the last 5 bp of *yddM* to ~1 kb downstream of *yddM* were used. For the ∆*ramT* mini-ICE, regions from ~1 kb upstream of the start of *ramT* to 12 bp after the end of *ramS*, from the end of *ramT* to the end of *ramA*, and from the last 5 bp of *yddM* to ~1 kb downstream of *yddM* were used. The kanamycin resistance cassette was obtained from pGK67 (2). These fragments were amplified using PCR, combined using linear isothermal assembly (63), and transformed into JH642, selecting for kanamycin resistance. Additional alleles (described above) were added to generate the final strains.

### UV irradiation and effects on cell growth

Cells were grown to mid-exponential phase (OD_600_ ~0.2) in defined minimal medium with 1% L-arabinose. D-xylose (1%) was added to activate ICE*Bs1* where indicated. After 15 min, 600 µL of each culture was placed into a sterile plastic petri dish, and samples were irradiated with UV light (5 J/m^2^) using a Stratalinker UV Crosslinker. Aliquots (180 µL) of each culture were then grown in a 96-well plate (96-Well Non-Treated Plates, GenClone) with three technical replicates for each culture. Cultures in the 96-well plates were grown at 37°C with continuous double orbital shaking at 807 cpm in a Biotek Synergy H1 plate reader and OD_600_ readings taken over the course of the experiment, typically for 10–12 h.

### β-Galactosidase assays

Cells were grown to mid-exponential phase (OD_600_ ~0.2) at 37°C in defined minimal medium with 1% L-arabinose. D-xylose (1%) was added to activate ICE*Bs1* where indicated. Samples were taken at indicated timepoints. Cells were permeabilized with 15 µL of toluene, and β-galactosidase-specific activity was determined [(ΔA420 per min per mL of culture per OD_600_ unit) × 1,000] essentially as described (67) after pelleting cell debris.

### Microscopy and image analysis

Imaging of single cells was done with an inverted microscope (Nikon, Ti-E) with a motorized stage and a CoolSnap HQ2 camera (Photometrics). A Nikon Intensilight mercury illuminator was used with appropriate sets of excitation and emission filters (filter set 49002 for GFP, Chroma). Cells were spotted on agarose pads set in a homemade incubation chamber made by stacking two sealable Frame-Seal Incubation Chambers (Bio-Rad). Agarose pads contained 1.5% Ultrapure Agarose (Thermo Fisher) dissolved in Spizizen salts medium (64).

Images and videos were processed with Fiji ImageJ (68). All images for fluorescent channels were subjected to background subtraction using 10 or 20 pixels (10 pixels were used for images with RecA-GFP filamentation). Cell meshes were acquired using Oufti (69). Meshes were analyzed in MATLAB using custom scripts.

Cells expressing mNeongreen were gated by comparing fluorescence to that of a strain containing the reporter but that did not contain ICE*Bs1* [ICE*Bs1*(0)]. Cells that had a fluorescence signal that was ≥3 standard deviations above the mean background fluorescence of cells with P*yneA-mNeongreen* and no ICE*Bs1* were considered to have activated the SOS response. In cells without ICE*Bs1*, this was <2% of cells in the population [Fig. 4, ICE(0)].
